# Tomato Yellow Leaf Curl Virus C4 Suppresses Antiviral Immunity by Targeting the 14‐3‐3 Protein SlTFT2

**DOI:** 10.1111/mpp.70337

**Published:** 2026-08-31

**Authors:** Danna Gao, Feng Sun, Xiaoxiao Gao, Yinghua Ji, Yuelin Zhu

**Affiliations:** ^1^ College of Horticulture Nanjing Agricultural University Nanjing China; ^2^ Institute of Plant Protection Jiangsu Academy of Agricultural Sciences Nanjing China

**Keywords:** 14‐3‐3 protein, C4, geminiviruses, nuclear accumulation, SlTFT2, TYLCV

## Abstract

Geminiviruses severely threaten global crop production. Tomato yellow leaf curl virus (TYLCV) encodes multifunctional effector C4, which participates in diverse biological processes and interacts with host proteins to facilitate infection. Despite extensive studies on geminiviral C4, how TYLCV C4 suppresses tomato immunity remains poorly characterized. In this study, we identified the tomato 14‐3‐3 protein SlTFT2 as a specific interaction partner of the TYLCV C4 protein. We demonstrated that *SlTFT2* functions as a positive regulator of antiviral defence, as its silencing enhanced systemic viral infection, whereas its overexpression restricted infection. A critical serine residue at position 86 in C4 was essential for this interaction, and mutation of this residue (S86A or S86L) in TYLCV infectious clones significantly attenuated viral infectivity. Furthermore, C4, but not the S86A or S86L mutants, reduced the nuclear accumulation of SlTFT2. Collectively, our findings establish that the C4–SlTFT2 interaction is indispensable for TYLCV‐mediated immunosuppression. Moreover, C4 promotes successful TYLCV infection by reducing the nuclear accumulation of SlTFT2 through this interaction, revealing a novel mechanism by which the virus subverts host defence by targeting a 14‐3‐3 protein.

## Graphical abstract

TYLCV C4 interacts with tomato 14‐3‐3 SlTFT2, a positive regulator of antiviral defence. C4 reduces SlTFT2 nuclear accumulation via this interaction, suppressing host immunity. Residue S86 is critical for this interaction; its mutation (S86A/S86L) attenuates viral infectivity, revealing a viral subversion mechanism.

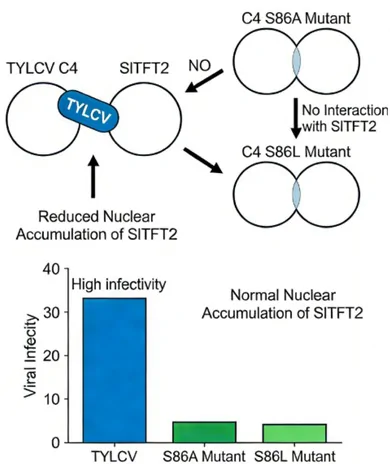

## 1 Introduction

Geminiviruses are a globally distributed group of plant viruses that infect a wide range of crops, causing severe diseases that result in substantial economic losses and pose a serious threat to food security. These viruses possess either monopartite (DNA‐A) or bipartite (DNA‐A and DNA‐B) single‐stranded circular DNA genomes, approximately 2.5–5.2 kb in size, which encode four to nine genes. Several of these genes are involved in pathogenicity and viral movement. Tomato yellow leaf curl virus (TYLCV), which is transmitted in a circulative manner by the whitefly *Bemisia tabaci* , represents one of the most serious constraints to tomato production worldwide (Delatte et al. 2007). TYLCV is a typical single‐stranded circular DNA virus belonging to the genus *Begomovirus* within the family *Geminiviridae*. It infects plants, leading to stunted growth, upward leaf curling and leaf yellowing. The TYLCV genome is widely recognized to contain six open reading frames (ORFs) (Fondong 2013; Li et al. 2022): the viral strand encodes V1 and V2, whereas the complementary strand encodes C1, C2, C3 and C4 (Fondong 2013). In addition, a recent study employing mass spectrometry analysis of TYLCV‐infected *Nicotiana benthamiana* leaves identified a novel C7 protein, which is proposed to be involved in viral transcription, movement and suppression of host RNA silencing (Liu et al. 2023).

The C4 protein of geminiviruses exemplifies the remarkable multifunctionality of geminiviral proteins (Kumar and Dasgupta 2023). Encoding a ~12 kDa protein, C4 is involved in viral movement (Jupin et al. 1994; Teng et al. 2010) and acts as a pathogenicity determinant (Latham et al. 1997; Luna et al. 2012; Mills‐Lujan and Deom 2010; Piroux et al. 2007). Significant progress has also been made in identifying host factors that interact with the TYLCV C4 protein (Dogra et al. 2009; Mei, Wang, et al. 2018; Mei, Yang, et al. 2018; Mei et al. 2021). Studies have demonstrated that the TYLCV C4 protein interacts with BARELY ANY MERISTEM 1 (BAM1), thereby suppressing the intercellular movement of RNA interference (RNAi) signals in *Arabidopsis* (Rosas‐Diaz et al. 2018), and disrupts chloroplast‐dependent antiviral salicylic acid (SA) biosynthesis (Medina‐Puche et al. 2020). Additional studies in *Arabidopsis* have revealed that TYLCV C4 engages in extensive interactions with plant receptor‐like kinases (Garnelo Gómez et al. 2019). Collectively, these studies demonstrate that the C4 protein plays a central role in disrupting plant defence responses (Ramesh et al. 2017; Kumar and Dasgupta 2023; Mei et al. 2023).

14‐3‐3 proteins are ubiquitous eukaryotic signalling components that are highly conserved from unicellular eukaryotes to higher plants and animals. These proteins form homo‐ or heterodimers through their N‐terminal domains, with each monomer containing a binding pocket for target proteins. Notably, both protomers within a 14‐3‐3 dimer can independently bind either two distinct target proteins or two binding sites within a single protein, enabling their function as molecular scaffolds or adaptors in diverse biological pathways (Braselmann and McCormick 1995; Yaffe et al. 1997). Their regulatory roles include enhancing or suppressing the catalytic activity of target proteins, modulating nuclear trafficking and subcellular localization, altering protein–protein interaction capabilities and protecting targets from proteolytic degradation (Cotelle et al. 2000; Sehnke et al. 2002; Aitken 2006; Latz et al. 2007; Ryu et al. 2007; Paul et al. 2012). Previous studies have demonstrated that plant 14‐3‐3 proteins participate in numerous biological processes, including primary metabolism, signal transduction, ion transport, transcriptional regulation and responses to both biotic and abiotic stresses (Ganguly et al. 2005; Schoonheim et al. 2007; Denison et al. 2011; Chen et al. 2017; Huang et al. 2022).

The involvement of 14‐3‐3 proteins in pathogen defence mechanisms has been well documented (Roberts et al. 2002), with accumulating evidence supporting their crucial roles in plant–pathogen interactions (Denison et al. 2011; Wilson et al. 2016; Rosas‐Diaz et al. 2018). Several 14‐3‐3 isoforms interact with the N protein in tobacco, which confers resistance to tobacco mosaic virus (Konagaya et al. 2004). Additionally, 14‐3‐3 proteins associate with RPW8.2, a resistance gene product that enhances powdery mildew resistance via the SA signalling pathway (Yang et al. 2009). These 14‐3‐3 proteins influence pathogen infection both by interacting with host defence‐related components and by serving as direct targets of pathogen effectors. For instance, the tomato 14‐3‐3 protein TFT7 interacts with SlMAPKKKα and SlMKK2 to positively regulate cell death (Oh et al. 2010; Oh and Martin 2011). However, the roles and underlying mechanisms of 14‐3‐3 proteins in viral infections—particularly those caused by DNA viruses—remain largely unclear.

In this study, we screened a tomato cDNA library and identified factors interacting with C4. Through this approach, SlTFT2, a member of the tomato 14‐3‐3 protein family, was identified as a potential target of C4. We subsequently confirmed and characterized the specific interaction between C4 and SlTFT2. Silencing of *SlTFT2* promoted systemic viral infection in tomato and *N. benthamiana*, whereas overexpression of *SlTFT2* inhibited viral infection. Moreover, we found that the C‐terminal region of C4 (amino acids 86–97) and the 14‐3‐3 domain of SlTFT2 are critical for their interaction. Mutation at position 86 of C4 impaired its interaction with SlTFT2, indicating that this residue is a key determinant of binding. Further analyses showed that mutation at position 86 also affected TYLCV infectivity. We constructed infectious clone vectors of TYLCV C4 mutants, TYLCV C4S86A and TYLCV C4S86L, mutations designed not to alter the codons of other overlapping viral proteins, which exhibited lower infectivity than TYLCV in host plants. These findings strongly suggest that the interaction between C4 and SlTFT2 is essential for TYLCV's immunosuppressive activity. Finally, when C4 was coexpressed with SlTFT2, C4 reduced the nuclear accumulation of SlTFT2, whereas the C4^S86A^ and C4^S86L^ mutants did not. This indicates that C4 may compromise the defensive function of SlTFT2 by reducing its nuclear accumulation through protein–protein interaction, thereby facilitating successful viral infection.

## 2 Results

### 2.1 TYLCV C4 Protein Interacts With Host SlTFT2

To elucidate the functional mechanism of the tomato yellow leaf curl virus (TYLCV) C4 protein during viral infection, we first performed affinity purification‐mass spectrometry screening in *N. benthamiana* and tomato to screen host candidate proteins interacting with C4, and we used luciferase complementation imaging (LCI) assay to screen and validate these candidate interactors (Figure S1). Among the candidates, a protein corresponding to the tomato *SlTFT2* gene (Solyc12g057110.2) was identified. The full‐length coding sequence of this candidate was amplified from tomato NJS cDNA using the specific primer pair SlTFT2‐BF/BR (Table S1), and a recombinant clone with the correct sequence was designated NJS‐1 for follow‐up experiments.

**FIGURE 1:**
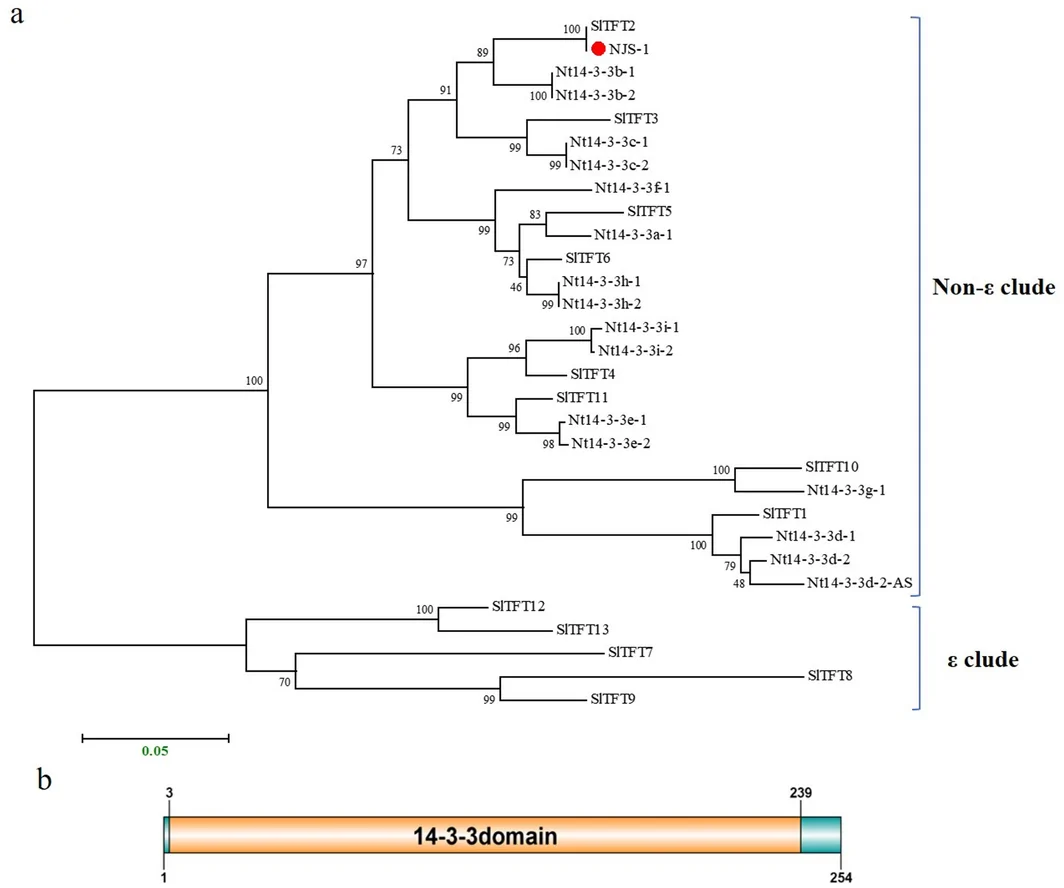
Phylogenetic analysis and domain architecture of *SlTFT2*. (a) Phylogenetic tree based on the full length nucleotide sequences of *SlTFT2* shown in Table S2. The nucleotide sequence alignment was performed in ClustalX2, and the phylogenetic tree was created with 1000 replicates by neighbour‐joining method in MEGA v. 11.0.9. Bootstrap scores over 90% were placed at major nodes. (b) Schematic diagram showing the SlTFT2 protein structure. 14‐3‐3 domain, numbers represent the amino acid positions of SlTFT2 protein.

To explore the evolutionary relationships of 14‐3‐3 family proteins, a phylogenetic tree was constructed in MEGA X using amino acid sequences retrieved from the Sol Genomics Network (SGN) for tomato and GenBank for *Nicotiana tabacum* . The tomato 14‐3‐3 family was clearly divided into two distinct clades: the *ε* group and the non‐ε group. Members including *SlTFT7*, *SlTFT8*, *SlTFT9*, *SlTFT12*, and *SlTFT13* clustered within the *ε* clade. The remaining SlTFT isoforms (*SlTFT1–SlTFT6*, *SlTFT10*, *SlTFT11*) belonged to the non‐ε clade and clustered closely with tobacco Nt14‐3‐3 homologues. Notably, *SlTFT2* exhibited the closest phylogenetic relationship with tobacco *Nt14‐3‐3b* (Figure 1a). Sequence information is provided in Table S2. InterPro analysis (https://www.ebi.ac.uk/interpro) predicted that SlTFT2 encodes a 254–amino acid protein containing a conserved 14‐3‐3 domain (Figure 1b).

To validate the interaction between C4 and SlTFT2, multiple independent assays were performed, including luciferase complementation imaging (LCI), bimolecular fluorescence complementation (BiFC), co‐immunoprecipitation (Co‐IP) and yeast two‐hybrid analyses (Y2H).

**FIGURE 2:**
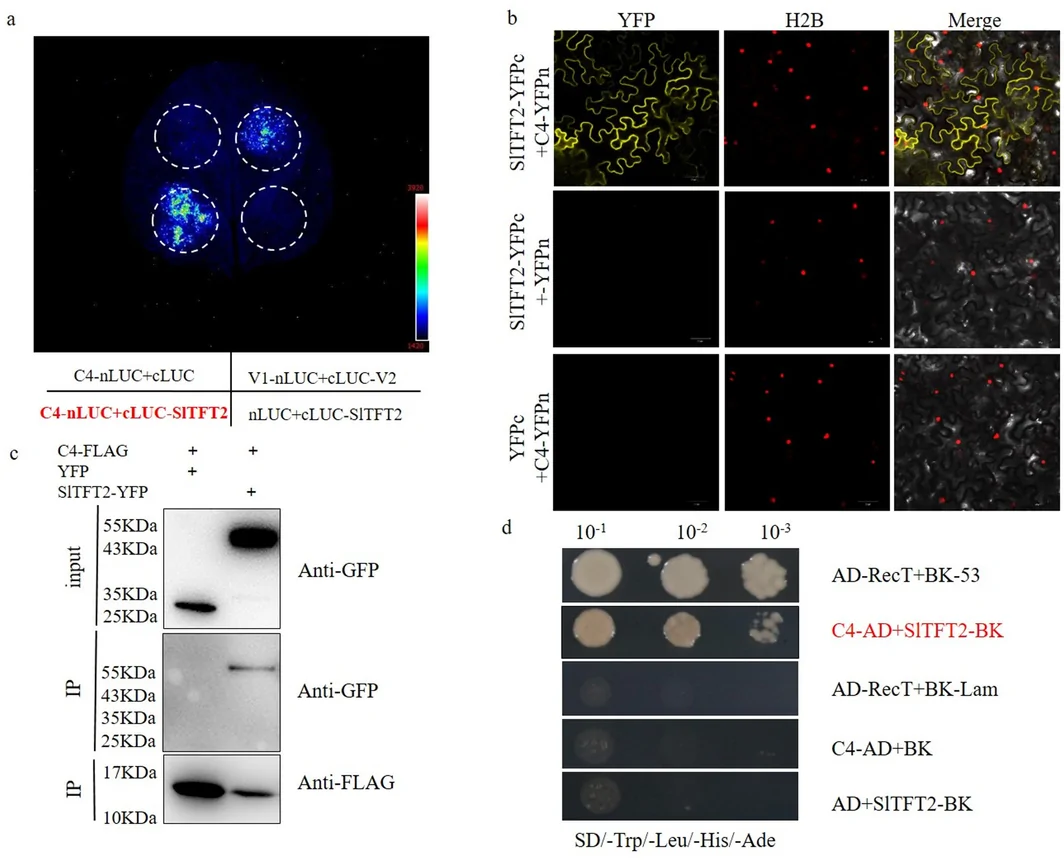
Tomato yellow leaf curl virus (TYLCV) C4 interacts with SlTFT2. (a) Luciferase complementation imaging (LCI) assays were performed to verify the interaction between TYLCV C4 and SlTFT2. nLUC and cLUC were used as empty vector controls. V1‐nLUC and cLUC‐V2 served as positive controls, as described previously. (b) For the bimolecular fluorescence complementation (BiFC) analysis of C4 interactions, *Nicotiana benthamiana* leaves were hand‐infiltrated with a suspension of two *Agrobacterium tumefaciens* GV3101 strains containing empty vectors alone, C4‐YFPn and SlTFT2‐YFPc, or empty vectors alone. Fluorescence was visualized using a confocal microscope (Zeiss LSM880) at an excitation wavelength (488 nm) 48 h after filtration. Bar, 50 μm. The experiment was repeated three times, with similar results. (c) Co‐immunoprecipitation (Co‐IP) assay to detect the interaction between C4‐FLAG and SlTFT2‐YFP. Crude extracts were prepared from *N. benthamiana* leaves infiltrated with *Agrobacterium* carrying C4‐FLAG, YFP or SlTFT2‐YFP. Total protein was incubated with an anti‐FLAG antibody, followed by immunoprecipitation with FLAG‐Trap beads. Western blot analysis was performed using antibodies against the FLAG tag and YFP tag to detect co‐immunoprecipitated proteins. The arrows indicate bands of the expected size (YFP 27 kDa; SlTFT2‐YFP 56.8 kDa; C4‐FLAG 12 kDa). (d) The interaction between C4 and SlTFT2 was assessed by yeast two‐hybrid (Y2H) assay. Yeast strains co‐transformed with the indicated plasmids were subjected to 10‐fold serial dilutions and grown on SD−Trp/−Leu/−His/−Ade medium. The strain‐carrying combinations of AD‐RecT/BK‐53 were used as positive controls, and those with AD‐RecT/BK‐Lam or empty vectors BK and AD were used as negative controls.

In the LCI assay, the full length sequence of *SlTFT2* was cloned into the pCAMBIA‐cLUC‐DC vector to generate the recombinant cLUC‐SlTFT2 construct. C4‐nLUC was co‐expressed with cLUC‐SlTFT2 in *N. benthamiana* leaves. Control combinations included C4‐nLUC or cLUC‐SlTFT2 paired with empty vectors. The previously reported interaction between TYLCV V1 and V2 served as a positive control (Zhao et al. 2018). A strong luminescence signal was observed specifically in leaves co‐expressing C4‐nLUC and cLUC‐SlTFT2, whereas no signal was detected in negative controls (Figure 2a).

For BIFC analysis, constructs encoding C4‐YFPn and SlTFT2‐YFPc were co‐infiltrated into *N. benthamiana* leaves. Confocal microscopy at 48 h post‐infiltration revealed distinct YFP fluorescence signals at the plasma membrane, confirming the interaction between C4 and SlTFT2 in planta (Figure 2b). No fluorescence was detected in control combinations.

Co‐IP assays further confirmed this interaction. C4‐FLAG was co‐expressed with SlTFT2‐YFP or YFP alone in *N. benthamiana* leaves. Immunoprecipitation with FLAG‐Trap beads followed by immunoblotting showed that SlTFT2‐YFP specifically co‐precipitated with C4‐FLAG, confirming the formation of C4–SlTFT2 protein complexes in planta (Figure 2c).

In Y2H assay, full‐length C4 fused to the activation domain and SlTFT2 fused to the binding domain enabled yeast growth on selective medium, comparable to the positive control. No growth was observed in negative control combinations (Figure 2d), demonstrating a direct interaction between C4 and SlTFT2.

### 2.2 The Transcription Level of *SITFT2* Is Regulated by TYLCV C4

**FIGURE 3:**
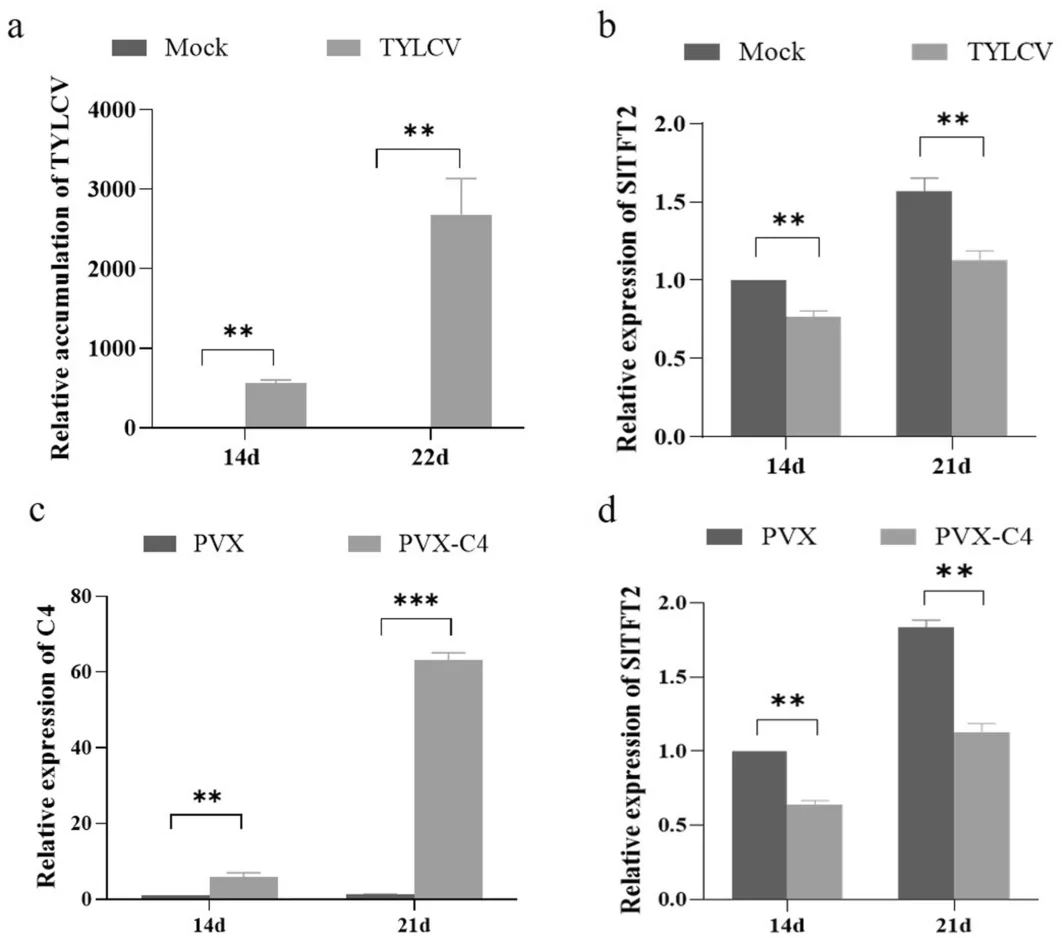
Tomato yellow leaf curl virus (TYLCV) C4 downregulates the transcriptional level of *SlTFT2*. (a) Quantitative PCR analysis of the relative accumulation TYLCV genomic DNA at 14 and 22 days post‐inoculation (dpi) in Mock‐ or TYLCV‐infected plant. *SlActin* was used as the reference gene. The *y*‐axis indicates the relative TYLCV genomic DNA accumulation. Errors bars represent SD of the mean. (b) Reverse‐transcription‐quantitative PCR (RT‐qPCR) analysis of the relative expression level of *SlTFT2* at 14 and 21 dpi in Mock‐ or TYLCV‐infected plant. (c) RT‐qPCR analysis of the relative expression level of TYLCV *C4* at 15 and 21 dpi in PVX‐ or PVX‐C4‐overexpressing plant. (d) RT‐qPCR analysis of the relative expression level of *SlTFT2* at 14 and 21 dpi in PVX‐ or PVX‐C4‐overexpressing plants. These experiments were repeated three times with similar results. Statistically significant difference (**p < 0.01, ***p < 0.001).

To investigate whether *SlTFT2* participates in TYLCV infection, tomato (cv. Moneymaker) plants were agroinoculated with TYLCV infectious clones. Quantitative analysis at 14 and 21 days post‐inoculation (dpi) revealed significantly elevated accumulation levels of TYLCV in virus‐inoculated plants compared to the empty vector (pBinPLUS) controls (Figure 3a), confirming successful viral infection. Notably, TYLCV‐infected systemic leaves exhibited reduced *SlTFT2* transcript accumulation relative to the controls (Figure 3b), indicating that TYLCV infection downregulates *SlTFT2* expression. Because TYLCV encodes the C4 protein that interacts with SlTFT2, we hypothesized that C4 could mediate this transcriptional suppression.

We constructed a C4 heterologous expression vector based on potato virus X (PVX), designated PVX‐C4. PVX is a viral vector widely used for transient heterologous expression of target proteins in tomato (Kong et al. 2013). We infiltrated the tomato plants and analysed the relative transcript levels of *SlTFT2* in the PVX (empty control)‐ and PVX‐C4‐treated tomato plants. At 14 and 21 dpi, the accumulation level of *C4* in the leaves of PVX‐C4‐treated tomato plants was significantly elevated (Figure 3c), indicating the successful overexpression of *C4* in tomato plants. The expression of the *SlTFT2* transcript in the PVX‐C4 plants was lower than that in the PVX‐treated control plants (Figure 3d), indicating that the C4 protein during TYLCV infection led to the downregulation of *SlTFT2*. During our sampling period at 14 and 21 dpi, only mild growth retardation was observed in PVX‐C4‐infected tomato plants, whereas no obvious leaf curling, yellowing or other visible abnormal phenotypes were detected in the two groups (Figure S2).

### 2.3 Silencing the *SlTFT2* Gene Increases the Susceptibility of Tomato Plants to TYLCV

**FIGURE 4:**
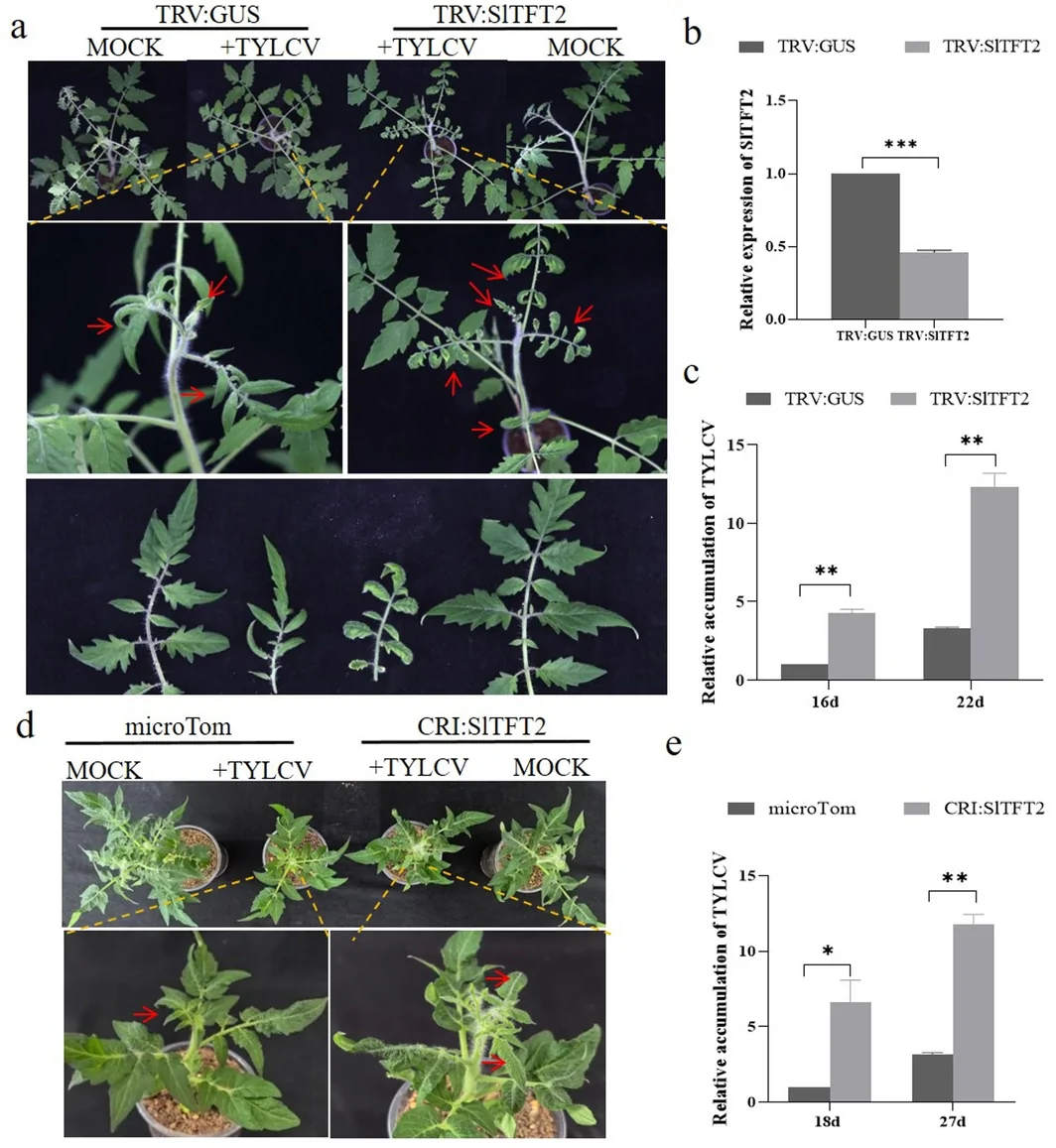
*SlTFT2*‐silenced plants were hypersusceptible to tomato yellow leaf curl virus (TYLCV) infection. (a) Symptoms caused by TRV‐mediated virus‐induced gene silencing with TRV:GUS or TRV:SlTFT2 following TYLCV infection or mock treatment as observed at 22 days post‐inoculation (dpi); a representative result is shown. Arrows highlight downward leaf curling symptoms. (b) The silencing efficiency of *SlTFT2* at 18 dpi as detected by reverse transcription‐quantitative PCR. (c) Quantitative PCR analysis of the relative accumulation of TYLCV at 16 and 22 dpi, TRV:GUS or TRV:SlTFT2 following TYLCV infection or mock treatment. *SlActin* was used as the reference gene. The *y*‐axis indicates relative TYLCV genomic DNA accumulation. (d) Symptomatic phenotypes of Micro‐Tom and Micro‐Tom plants with knockout of *SlTFT2* (CRISPR‐SlTFT2) plants infected by TYLCV. Photographs were taken at 18 dpi; a representative result is shown. Arrows highlight downward leaf curling symptoms. (e) Quantitative PCR analysis of the relative accumulation of TYLCV at 18 and 27 dpi. These experiments were repeated three times with similar results. Statistically significant differences (*p < 0.05, **p < 0.01, ***p < 0.001).

To better understand the function of *SlTFT2* in plants and its potential influence on viral infection, we silenced *SlTFT2* in tomatoes using a tobacco rattle virus (TRV)‐based virus‐induced gene silencing (VIGS) system. A 300 bp specific coding fragment of *SlTFT2* screened from the SGN VIGS database was inserted into the pTRV2 vector to generate the recombinant construct pTRV2:SlTFT2. Sequence alignment confirmed the high specificity of this silencing fragment with negligible off‐target effects on other 14‐3‐3 family members (Figure S3). *Agrobacterium tumefaciens* carrying recombinant pTRV2‐VIGS constructs (pTRV2:SlTFT2, pTRV2:GUS or pTRV2:PDS) were mixed with *Agrobacterium* carrying pTRV1 and inoculated into tomato plants. At 17 days post‐infiltration (dpi), obvious photobleaching was observed in TRV:PDS‐silenced plants. Plants inoculated with TRV:SlTFT2 exhibited only mild leaf curling in the upper leaves and appeared to grow normally 17 dpi (Figure S4). Subsequently, TYLCV was inoculated separately into the TRV:GUS and TRV:SlTFT2 plants. After *Agrobacterium*‐mediated infection with TYLCV, *SlTFT2*‐silenced plants displayed more severe symptoms, such as yellowing and leaf curling, than the control plants (Figure 4a). RT‐qPCR analysis revealed that *SlTFT2* expression in TRV:SlTFT2 plants was significantly lower than that in TRV:GUS plants at 17 dpi (Figure 4b), confirming successful silencing of *SlTFT2* in tomatoes. Additionally, qPCR results showed that the relative accumulation of TYLCV in the systemic leaves of TRV:SlTFT2‐treated plants was higher than that in the control plants at 14 and 21 dpi (Figure 4c). These findings suggest that silencing *SlTFT2* enhances tomato susceptibility to TYLCV infection, indicating that *SlTFT2* may act as a disease resistance factor and play a crucial role in disease symptom development and viral accumulation through the interaction between SlTFT2 and TYLCV C4.

To validate these findings, we generated *SlTFT2* knockout lines in tomato plants (cv. ‘Micro‐Tom’) using CRISPR/Cas9 technology (Figure S5). After inoculating both wild‐type (WT) and CRISPR‐SlTFT2 plants with TYLCV, at 18 days, the CRISPR‐SlTFT2 plants exhibited downward‐curling leaf symptoms (Figure 4d). As expected, qPCR analysis revealed that the relative accumulation of TYLCV in the systemic leaves of CRISPR‐SlTFT2 inoculated plants was higher than that in control plants at both 18 and 27 dpi (Figure 4e).

### 2.4 Overexpression of the *SlTFT2* Gene Inhibits TYLCV Infection

**FIGURE 5:**
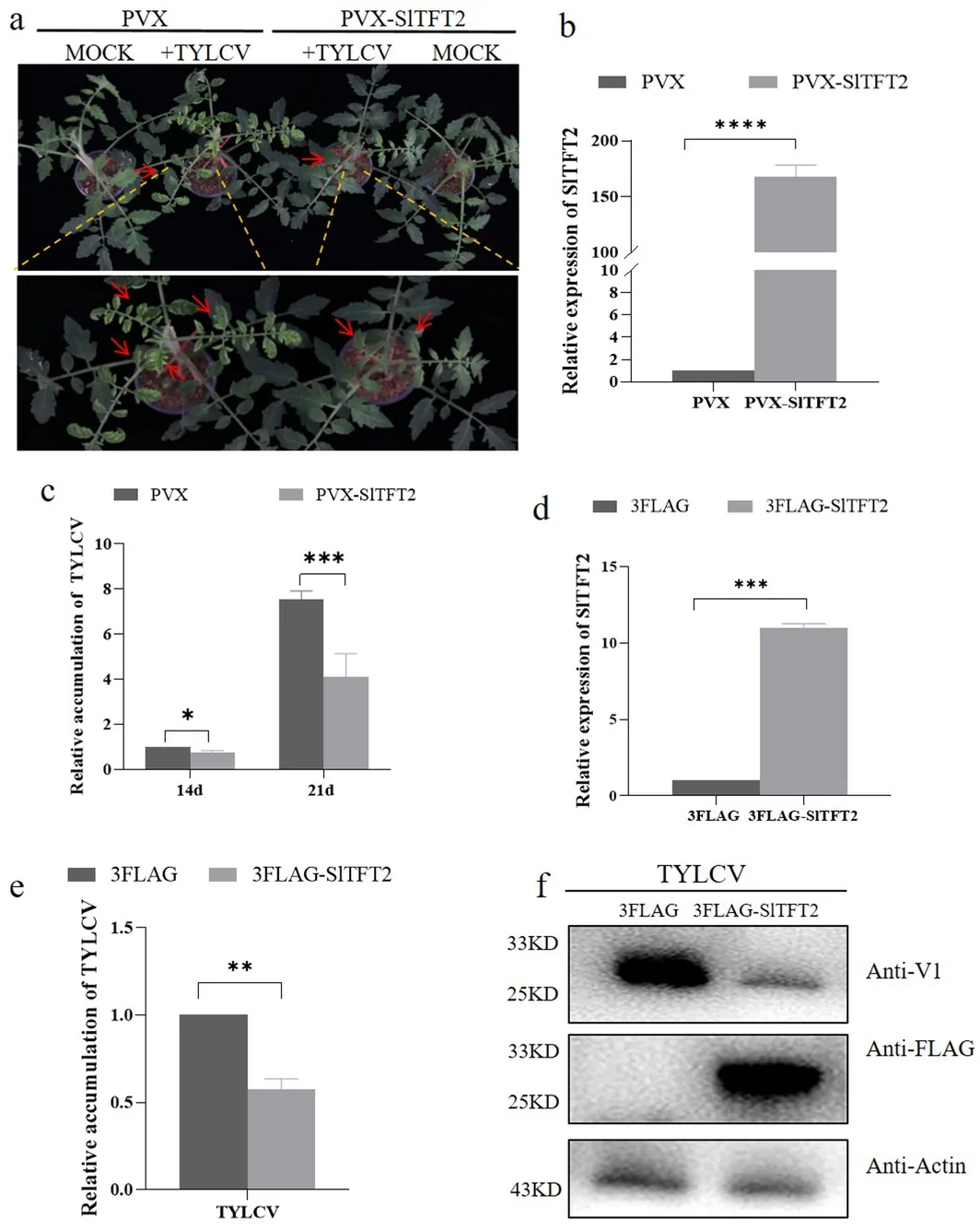
Overexpression of *SlTFT2* impedes tomato yellow leaf curl virus (TYLCV) infection. (a) Phenotypes of PVX and PVX‐SlTFT2 plants infected by TYLCV or mock treatment. Photographs were taken at 21 days post‐inoculation (dpi); a representative result is shown. (b) Reverse transcription‐quantitative PCR (RT‐qPCR) analysis of the relative expression level of *SlTFT2* at 8 dpi. (c) Quantitative PCR analysis of the relative accumulation of TYLCV at 14 and 21 dpi. (d) RT‐qPCR analysis of the relative expression level of *SlTFT2* at 48 h, in 3FLAG or 3FLAG‐SlTFT2 plants. (e) Quantitative PCR analysis of the relative accumulation of TYLCV at 48 h. (f) The accumulation of TYLCV coat protein (V1) in 3FLAG‐SlTFT2 and 3FLAG leaves was analysed by western blotting (d) with an anti‐TYLCV V1 antibody. These experiments were repeated three times with similar results. Statistically significant difference (*p < 0.05, **p < 0.01, ***p < 0.001, ****p < 0.0001).

The overexpression vector PVX‐SlTFT2 was constructed, and both PVX‐SlTFT2 and its empty control PVX were inoculated onto tomato plants to observe any symptoms (Figure S6). After agroinoculating tomato plants with TYLCV, we observed at 14 dpi that *SlTFT2*‐overexpressing tomato plants developed milder disease symptoms compared with control plants (Figure 5a). We measured *SlTFT2* expression levels via RT‐qPCR and found that *SlTFT2* expression was significantly higher in tomato plants inoculated with PVX‐SlTFT2 than PVX, indicating successful overexpression of *SlTFT2* (Figure 5b). Additionally, qPCR results after TYLCV inoculation showed that the relative accumulation of TYLCV in *SlTFT2*‐overexpressing plants was lower than that in the control plants at 14 and 21 dpi, suggesting that overexpression of *SlTFT2* inhibits TYLCV infection (Figure 5c).

To further validate whether *SlTFT2* overexpression inhibits TYLCV infection, we constructed a transient overexpression vector, pCambia2300:35S × 3FLAG‐SlTFT2 (hereafter referred to as 3FLAG‐SlTFT2). We transiently co‐expressed pCambia2300:35S × 3FLAG (hereafter referred to as 3FLAG) or 3FLAG‐SlTFT2 with TYLCV and leaf samples were collected 48 h after infiltration for qPCR and total protein extraction assays. As shown in Figure 5d, the expression level of *SlTFT2* in 3FLAG‐SlTFT2 cells was significantly higher than that in 3FLAG cells. The relative expression level of TYLCV in leaves co‐infiltrated with 3FLAG‐SlTFT2 and TYLCV was notably reduced (Figure 5e). Western blot analysis yielded consistent results, and the V1 protein level in TYLCV‐infected leaves co‐expressed 3FLAG‐SlTFT2 was lower than that in the control, indicating that overexpression of *SlTFT2* suppressed TYLCV infection (Figure 5f).

### 2.5 The C‐Terminal of C4 and the 14‐3‐3 Domain of SlTFT2 Are the Key Regions for Their Interaction, With the 86th Amino Acid of C4 Being a Critical Site

**FIGURE 6:**
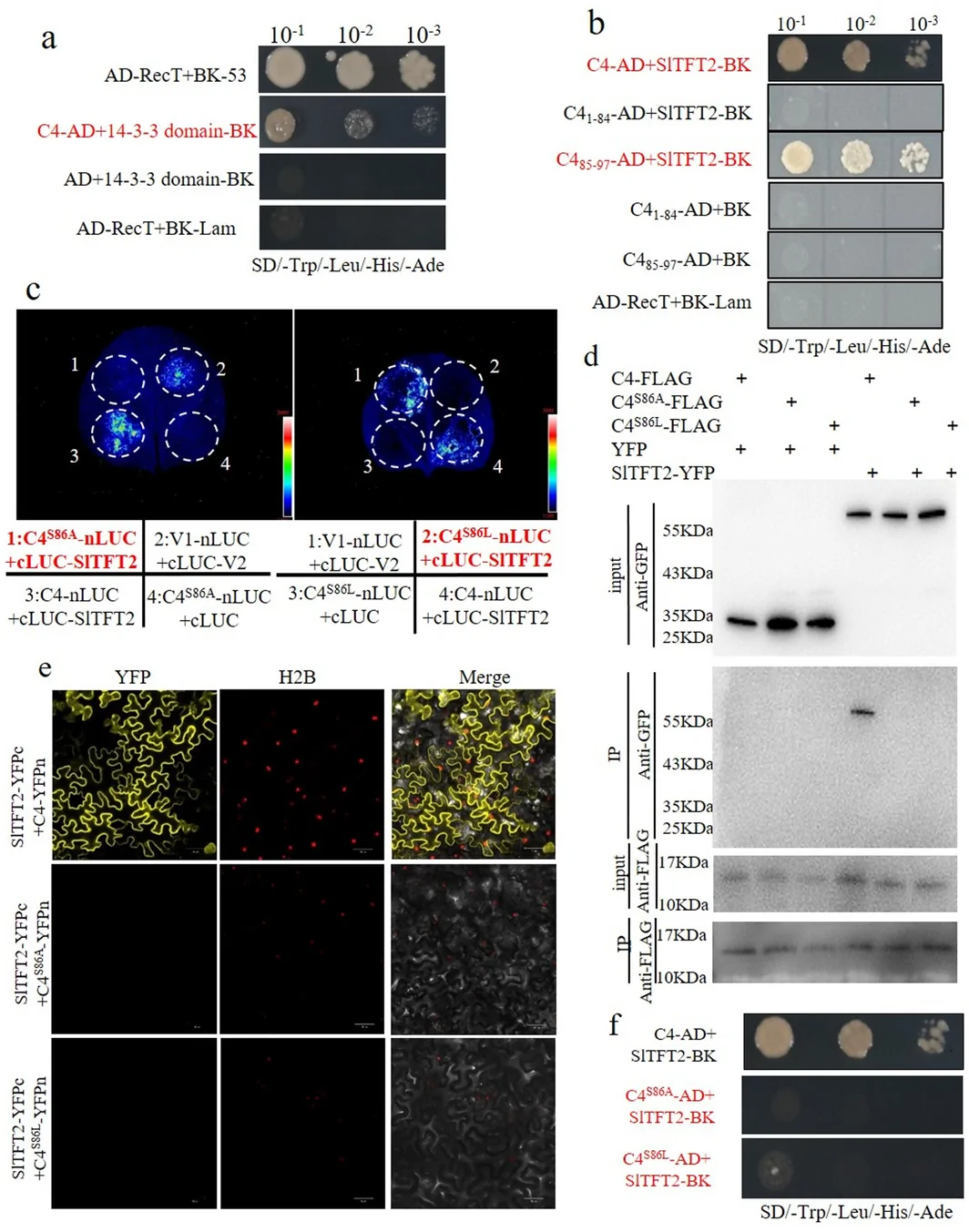
The C‐terminus of C4 and 14‐3‐3 domain of SlTFT2 are the key regions for the interaction, with Ser86 in C4 identified as the key residue mediating this association. (a) Identification of the interaction between TYLCV C4 and 14‐3‐3 domain of SlTFT2 in yeast. *Saccharomyces cerevisiae* AH109 was co‐transformed with the indicated plasmids and subjected to a 10‐fold series dilution and grown on SD/−Leu/−Trp/−His/−Ade medium. (b) Identification of the interaction between TYLCV C4 (85–97 amino acids) segment and SlTFT2 in yeast. TYLCV C4 (1–84 amino acids) segment had no interaction with SlTFT2. (c) Luciferase complementation imaging (LCI) assay demonstrated that the TYLCV C4 mutant (S86A and S86L) lost its interaction with the SlTFT2 protein. nLUC and cLUC are empty vector controls. The TYLCV C4 and SlTFT2 are positive controls. (d) Co‐immunoprecipitation (Co‐IP) assay to show the TYLCV C4 mutants (S86A and S86L) lost the interaction with the SlTFT2 protein. Crude extracts were prepared from *Nicotiana benthamiana* leaves infiltrated with *Agrobacterium tumefaciens* carrying C4‐FLAG, C4^S86A^‐FLAG, C4^S86L^‐FLAG and YFP or SlTFT2‐YFP. (e) Bimolecular fluorescence complementation (BiFC) analysis of TYLCV C4 mutants (S86A and S86L) interactions. (f) Yeast two‐hybrid (Y2H) assay showed that the C4 mutants do not interact with SlTFT2. A strain carrying the combination of C4‐AD/SlTFT2‐BK was used as a positive control.

To identify the key region of interaction, we constructed a 14‐3‐3 domain‐BK vector based on the 14‐3‐3 domain of SlTFT2 and performed a Y2H assay with C4‐AD (AD, activation domain vector pGADT7; BK, bait vector pGBKT7). The results showed that C4‐AD interacted with 14‐3‐3 domain‐BK (Figure 6a), indicating that the 14‐3‐3 domain is essential for the interaction.

We next generated truncated C4 constructs C4_1–84_‐AD and C4_85–97_‐AD for Y2H analysis. When co‐transformed with SlTFT2‐BK into *Saccharomyces cerevisiae* AH109, C4_85–97_‐AD, but not C4_1–84_‐AD, interacted with SlTFT2 (Figure 6b), identifying the C‐terminal region (85–97 amino acids [aa]) of C4 as critical for binding.

Next, site‐directed mutagenesis was performed to identify the key residues involved in this interaction. Given that 14‐3‐3 proteins specifically recognize phosphorylated serine/threonine motifs (Liu et al. 2021), we first analysed the amino acid sequence of the C4 fragment (85–97 aa) responsible for binding SlTFT2. Only two serine residues (Ser86 and Ser95) were found within this interacting region (Figure S7a). We constructed both S86A and S95A substitution mutants for parallel interaction verification. LCI detection revealed that the S95A mutation exerted no influence on the C4–SlTFT2 interaction (Figure S7b). The experimental results showed that no luminescence was observed with nLUC‐C4^S86A^ and cLUC‐SlTFT2 (Figure 6c), while the positive control (nLUC‐C4 with cLUC‐SlTFT2) exhibited normal luminescence. This was further validated by Co‐IP assays (Figure 6d). The results from the BiFC assay (Figure 6e) and Y2H assay (Figure 6f) also corroborated these findings. This indicates that the S86 site in C4 is critical for its interaction with SlTFT2.

### 2.6 C4 Protein Mutation Impairs TYLCV Infectivity

**FIGURE 7:**
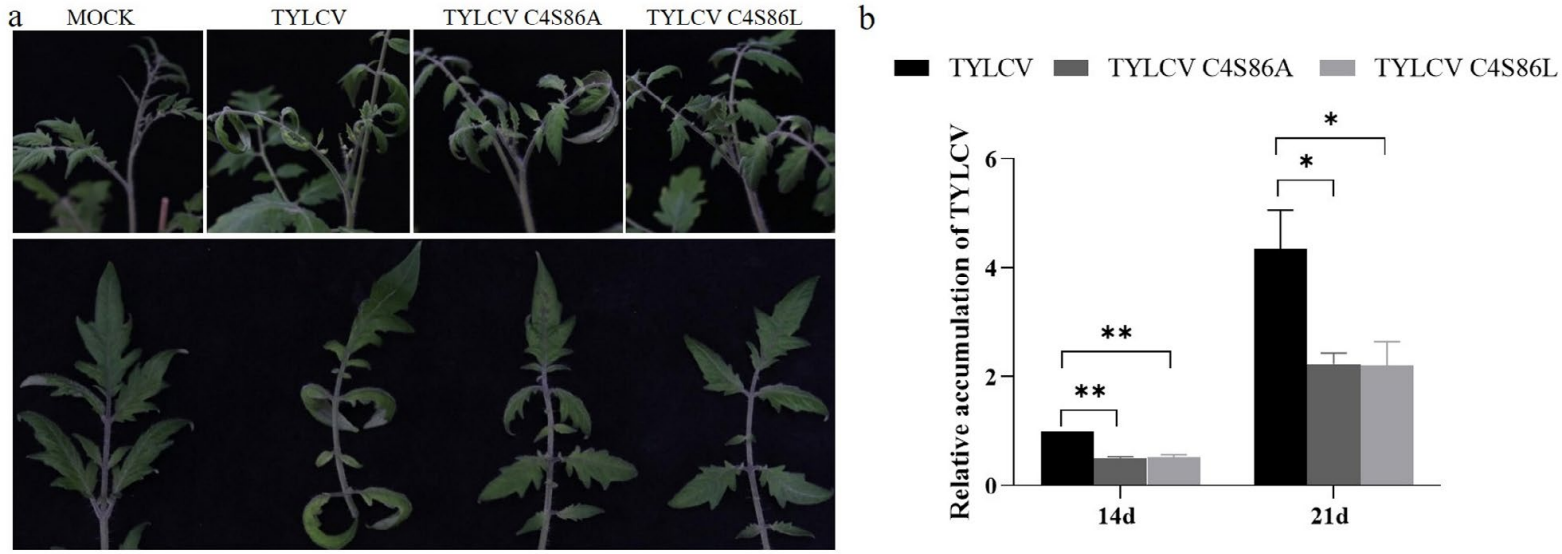
C4 protein mutation impairs tomato yellow leaf curl virus (TYLCV) infectivity. (a) Phenotype of tomato plants inoculated with TYLCV infectious clones producing C4, SlTFT2‐interaction‐compromised C4 mutants S86A or S86L (mutations that do not alter other overlapping viral proteins) at 23 days post‐inoculation (dpi). A representative result is shown. (b) Quantitative PCR analysis of the relative accumulation of TYLCV at 14 and 21 dpi. Statistically significant difference (*p < 0.05, **p < 0.01).

To clarify the effect of S86A of C4 on viral pathogenicity, we constructed infectious clones of mutant viruses. The C4 point mutations S86A and S86L were introduced into the pBinPLUS vector to generate TYLCV C4S86A and TYLCV C4S86L. Of note, the S86L mutation only alters the C4 protein, with no effect on the C1 protein encoded by the complementary strand of the TYLCV genome; this mutant has been confirmed to lose interaction with SlTFT2. Tomato plants were inoculated with infectious clones of TYLCV or TYLCV C4S86A and TYLCV C4S86L. Observations showed that at 13–14 dpi, TYLCV developed curling symptoms earlier than the mutants. In contrast, both TYLCV C4 mutants began to show symptoms at approximately 18 dpi, and the symptoms were milder than TYLCV (Figure 7a). The qPCR results revealed that, at 14 and 21 dpi, the relative accumulation of the TYLCV in tomatoes inoculated with TYLCV C4S86A and TYLCV C4S86L infectious clones was lower than that in the TYLCV (Figure 7b), indicating that mutations at this site impair the infection efficiency of TYLCV in tomatoes.

### 2.7 C4 Protein Reduces Nuclear Accumulation of SlTFT2

**FIGURE 8:**
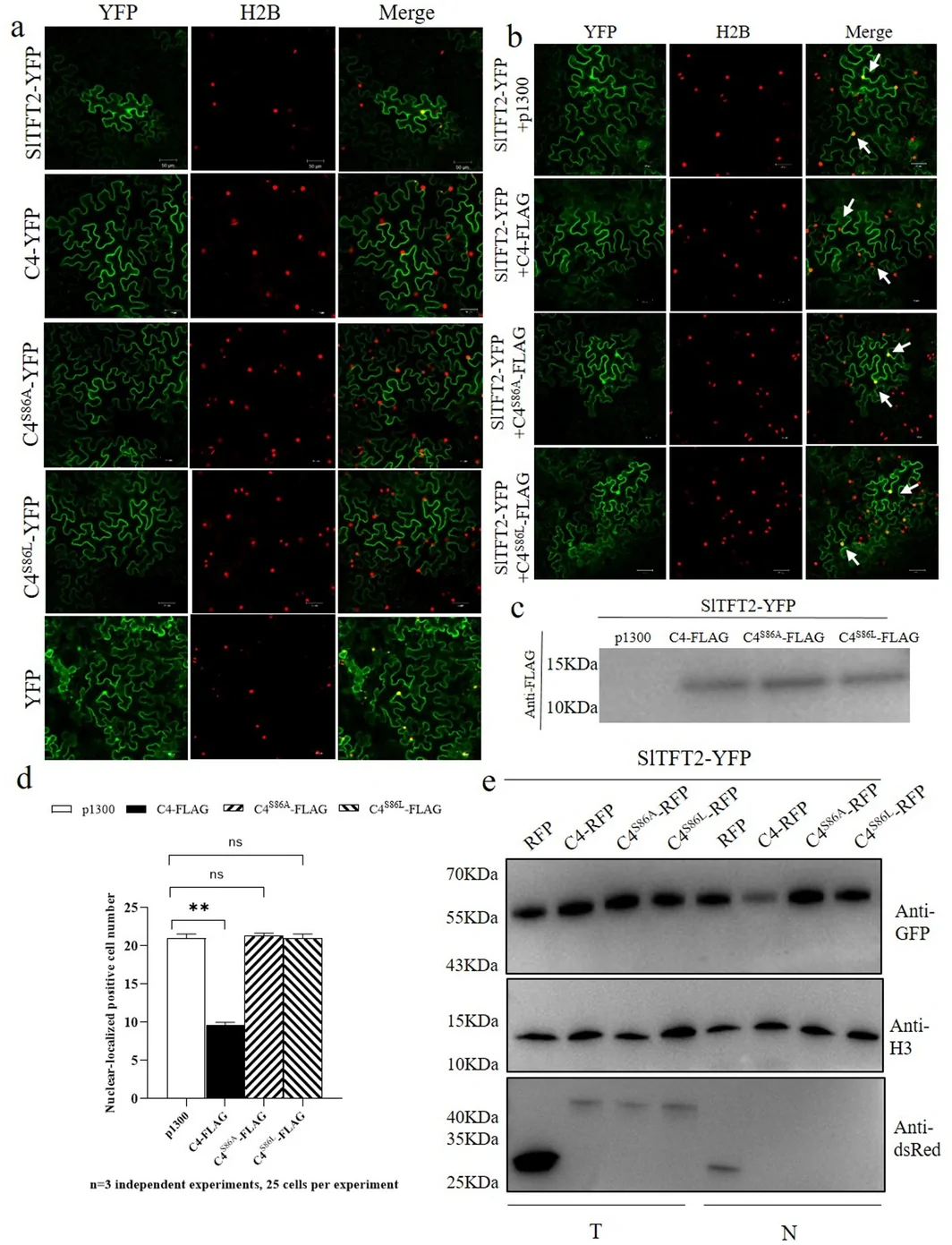
C4 protein reduces nuclear accumulation of SlTFT2. (a) Subcellular localization of SlTFT2‐YFP, C4‐YFP, C4^S86A^‐YFP or C4^S86L^‐YFP fusion protein in *Nicotiana benthamiana* epidermal cells in the presence of H2B‐RFP. Confocal micrographs were taken at 48 h post‐infiltration (hpi). Bars, 50 μm. (b) Distribution patterns of SlTFT2‐YFP in the presence of p1300, C4‐FLAG, C4^S86A^‐FLAG or C4^S86L^‐FLAG expressed in the *N. benthamiana* epidermal cells. Arrows indicate the nuclear localization of SlTFT2‐YFP fluorescence signals in merged images. Scale bar: 50 μm. (c) Western blot analysis confirming the normal expression of C4‐FLAG, C4^S86A^‐FLAG, and C4^S86L^‐FLAG in leaf tissues. (d) Effect of TYLCV C4 and its mutants on the nuclear localization of SlTFT2‐YFP. Data are presented as the mean of three independent experiments, with 25 cells counted per experiment. Statistically significant difference (**p < 0.01). (e) Nuclear‐cytoplasmic fractionation assays performed. T represents total protein, N represents nuclear protein. Histone H3 served as the nuclear protein marker.

To further investigate how C4 targets SlTFT2 to enable successful TYLCV infection, we amplified the genes encoding C4, C4 mutants, and SlTFT2. After amplification and digestion (with BamHI), C4, C4S86A, and C4S86L were cloned into the pCambia1300–YFP vector. After sequence verification, C4‐YFP, C4^S86A^‐YFP, and C4^S86L^‐YFP and SlTFT2‐YFP were obtained. The recombinant YFP vectors were transformed into *Agrobacterium* and co‐infiltrated with H2B‐RFP into *N. benthamiana* leaves. After 48 h, the fluorescence was observed using a confocal laser‐scanning microscope. C4, C4^S86A^ and C4^S86L^ were all localized to the plasma membrane at the subcellular level, indicating that the mutation at S86 in C4 did not alter its subcellular localization. SlTFT2 was localized to both the plasma membrane and nucleus at the subcellular level (Figure 8a). YFP alone, which was used as a control, localized in the plasma membrane and nucleus. H2B‐RFP, which was employed as a nuclear marker, was specifically localized to the nucleus.

As demonstrated by previous BiFC confocal microscopy assays, the interaction signal between SlTFT2‐YFPc and C4‐YFPn was predominantly detected at the plasma membrane, with virtually no signal observed in the nucleus (Figure 2b). The fluorescence distribution pattern of the C4–SlTFT2 interacting complex matched that of free C4‐YFP, but deviated distinctly from the localization profile of free SlTFT2‐YFP. These results suggested that co‐expression of TYLCV C4 is capable of altering the subcellular distribution of SlTFT2‐YFP.

To verify this hypothesis, the coding sequences of C4 and its mutants were digested with the single restriction enzyme BamHI and inserted into the p1300‐RFP vector to generate recombinant C4‐RFP, C4^S86A^‐RFP and C4^S86L^‐RFP constructs. The SlTFT2‐YFP plasmid was transformed into *A. tumefaciens* , followed by leaf co‐infiltration with empty RFP, C4‐RFP, C4^S86A^‐RFP or C4^S86L^‐RFP individually. Leaf tissues were harvested at 48 hpi for co‐localization analysis. In leaves co‐expressing SlTFT2‐YFP and empty RFP, fluorescence signals were detected at both the plasma membrane and nucleus. For plants co‐infiltrated with SlTFT2‐YFP together with C4‐RFP, C4^S86A^‐RFP or C4^S86L^‐RFP, co‐localization signals were observed at the plasma membrane in all combinations (Figure S8). Notably, co‐expression with C4‐RFP markedly reduced nuclear accumulation of SlTFT2‐YFP. However, this inhibitory phenotype was fully abolished in leaves co‐infiltrated with C4^S86A^‐RFP or C4^S86L^‐RFP.

To further verify the above observations, SlTFT2‐YFP was co‐expressed with C4‐FLAG, C4^S86A^‐FLAG or C4^S86L^‐FLAG. Empty vector pCambia1300 (p1300) was used as a negative control. Confocal microscopy results after 48 h showed that when SlTFT2‐YFP was co‐infiltrated with p1300, SlTFT2 protein was present both in the plasma membrane and in the nucleus. However, when SlTFT2‐YFP was co‐expressed with C4‐FLAG, the nuclear signal of SlTFT2‐YFP decreased. In contrast, co‐expression of SlTFT2‐YFP with either C4^S86A^‐FLAG or C4^S86L^‐FLAG resulted in SlTFT2 protein being detected in both the plasma membrane and nucleus, with no reduction in nuclear expression (Figure 8b). These results indicated that C4 reduced the proportion of SlTFT2 localized to the nucleus. Western blotting confirmed the successful expression of C4‐FLAG, C4^S86A^‐FLAG and C4^S86L^‐FLAG in leaves (Figure 8c). We selected 25 YFP‐positive cells with intact cell morphology from different fields of view and the experiment was repeated three times. Confocal microscopy showed that the percentage of cells with SlTFT2 nuclear localization was approximately 88% in the control and mutant groups (p1300 + SlTFT2‐YFP, C4^S86A^‐FLAG + SlTFT2‐YFP or C4^S86L^‐FLAG + SlTFT2‐YFP), whereas it was only 40% in the C4‐expressing group (C4‐FLAG + SlTFT2‐YFP) (Figure 8d). This demonstrates that C4 significantly inhibits the nuclear localization of SlTFT2.

To further verify our hypothesis, we conducted nuclear‐cytoplasmic fractionation experiments via co‐infiltration of SlTFT2‐YFP with RFP, C4–RFP, C4^S86A^–RFP or C4^S86L^–RFP. Leaf samples were collected at 48 hpi to examine whether C4 could reduce the nuclear enrichment of SlTFT2 inside plant cells. Histone H3 was used as a nuclear reference protein and immunodetected with anti‐H3 antibody. The results showed that in the presence of C4–RFP, the expression level of SlTFT2 in the nucleus was significantly lower than that in the other three groups (Figure 8e). This further indicated that the presence of C4 reduced the nuclear expression of SlTFT2, whereas the mutant form did not reduce SlTFT2 nuclear accumulation. This further demonstrates that the interaction between C4 and SlTFT2 is essential for the C4‐mediated relocalization of SlTFT2.

## 3 Discussion

In the arms race between geminiviruses and their plant hosts, plants have evolved effective strategies targeting various stages of viral infection. In response, geminiviruses encode multifunctional proteins such as C4, which hijack specific host factors to subvert plant defence mechanisms (Lai et al. 2009). The C4 protein can target multiple host proteins, including Kip‐related proteins (KRPs), BARELY ANY MERISTEM 1 (BAM1), and BRI1 KINASE INHIBITOR 1 (BKI1), to facilitate viral infection (Li et al. 2020; Mei et al. 2021). In the present study, we have identified a novel interacting partner of the TYLCV C4 protein: SlTFT2, a member of the tomato 14‐3‐3 protein family. The specific interaction between C4 and SlTFT2 was experimentally validated using a comprehensive approach, including firefly LCI, BiFC, Co‐IP and Y2H assays. Following TYLCV infection, the expression level of the *SlTFT2* gene was significantly downregulated. Functional analysis revealed that silencing *SlTFT2* promoted viral infection, while overexpression of *SlTFT2* inhibited viral infection. These findings indicate that SlTFT2 acts as a negative regulator during TYLCV infection and is involved in tomato's resistance mechanism against the virus. Further investigations into interaction domains revealed that the 14‐3‐3 domain of SlTFT2 is critical for mediating their interaction. As 14‐3‐3 proteins act as immune hubs in plants, they are commonly targeted by pathogenic effectors (Zhao et al. 2021). In recent years, the role of 14‐3‐3 proteins in plant antiviral resistance has garnered increasing attention, although reported studies have predominantly focused on RNA viruses. For instance, the coat protein of beet black scorch virus (BBSV) targets 14‐3‐3 proteins to disrupt MAPKKKα‐mediated immunity in plants (Gao et al. 2022). Nb14‐3‐3 h mediates antiviral defence against potato virus Y (PVY) infection by sequestering NbTCTP (Fang et al. 2024). In maize, ZmGF14‐6 is a key factor in suppressing sugarcane mosaic virus (SCMV) infections in maize plants (Xu et al. 2025). These findings collectively indicate that 14‐3‐3 proteins serve as critical targets for successful RNA virus infection while also playing essential roles in plant defence against RNA viruses. In contrast, this study focuses on a single‐stranded circular DNA virus, TYLCV, further substantiating the broad importance of 14‐3‐3 proteins in plant immunity and demonstrating their involvement in defence against DNA viruses. However, whether the underlying defence mechanisms are conserved requires further investigation.

Specifically, the S86 residue in C4 was identified as a core site essential for regulating this interaction. To elucidate the function of the Ser86 residue within C4, we constructed infectious clones of TYLCV C4S86 mutants for plant infection assays. The results demonstrated that, compared to the TYLCV, infection with the C4 S86 site mutants led to attenuated symptom development and a significant reduction in viral accumulation in tomato plants. This indicates that the pathogenicity of the TYLCV C4 mutants S86A and S86L was compromised. However, whether this observed decrease in infectivity is directly related to the impaired interaction with SlTFT2 requires further investigation. Both C4 and its S86 site mutants (C4S86A, C4S86L) localized to the cell membrane, indicating that mutations at the C4 S86 site did not alter its inherent subcellular localization pattern. Previous results demonstrated that SlTFT2 localizes to the plasma membrane and nucleus. Co‐expression experiments of C4 or its S86 mutants with SlTFT2 showed that C4 significantly altered the subcellular distribution of SlTFT2, specifically manifesting as a marked reduction in nuclear fluorescence signal of SlTFT2. In contrast, no such relocalization was observed when SlTFT2 was co‐expressed with C4 S86 mutants. This finding was further corroborated by nuclear‐cytoplasmic fractionation assays: upon co‐expression of C4‐RFP with SlTFT2‐YFP, the nuclear accumulation of SlTFT2 was significantly reduced compared to the control; however, co‐expression of either C4^S86A^‐RFP or C4^S86L^‐RFP with SlTFT2‐YFP did not decrease the nuclear level of SlTFT2. Previous research has demonstrated that TLCYnV C4 alters the localization pattern of NbSKη, recruiting it to the membrane and reducing its nuclear accumulation (Mei, Wang, et al. 2018; Mei, Yang, et al. 2018). The C4 protein of sweet potato leaf curl virus‐Jiangsu (SPLCV‐JS) interacts with AtBIN2, leading to the re‐localization of the AtBIN2‐interacting proteins AtBES1/AtBZR1 into the nucleus, thereby activating the brassinosteroid signalling pathway (Bi et al. 2017). TYLCV C4 outcompetes endogenous interactors of RLDs, hijacking RLD proteins to the plasma membrane and disrupting their functions in coordinating endomembrane trafficking and polar auxin transport, which ultimately contributes to the development of viral symptoms (Gao et al. 2025). Compared with these studies, the present work is the first to clarify the regulatory effect of a geminiviral C4 protein on the subcellular localization of SlTFT2. This finding supplements the molecular mechanism by which 14‐3‐3 proteins, as host targets, are involved in DNA virus infection. It further enriches the molecular strategies employed by geminiviral C4 proteins, which achieve efficient infection by reprogramming the subcellular localization of host factors and thereby interfering with normal host physiological functions. Our study demonstrates that C4 reduces the nuclear accumulation of SlTFT2; however, the specific organelle to which SlTFT2 is relocated remains unclear and will be the subject of future investigation.

While the molecular details of many 14‐3‐3 interactions remain incompletely understood, it appears that several of its binding substrates perform critical functions within the nucleus, or their ability to shuttle between the nucleus and cytoplasm is essential. In plants, Repression of shoot growth (RSG) continuously shuttles between the nucleus and cytoplasm, and this intracellular compartmentalization of RSG may play a role in the tightly regulated biosynthesis of gibberellins in plants (Igarashi et al. 2001). Brassinosteroid‐induced nuclear localization is strongly associated with the dephosphorylation of BZR1 and the inhibition of its binding to 14‐3‐3 proteins (Gampala et al. 2007). The phosphorylation of MYBS2 is crucial for regulating its sugar‐dependent nucleocytoplasmic shuttling and interaction with 14‐3‐3 proteins, representing a regulatory mechanism that modulates gene expression in response to sugar status (Chen et al. 2019). Tobacco DBP1 specifically interacts with 14‐3‐3G, and this interaction mediates the nucleocytoplasmic shuttling of DBP1, positively regulating the induction of the *CEVI1* gene in tobacco (Carrasco et al. 2006). In the present study, TYLCV C4 interacts with the 14‐3‐3 protein SlTFT2, thereby modulating its nuclear localization. This implies that C4 likely disrupts the normal function of SlTFT2 in the nucleus, potentially further impairing its stable nucleocytoplasmic shuttling or its normal roles as a signal transducer or molecular chaperone. Once interfered with by the virus, this process would directly impact host immune responses or growth and developmental regulation.

TYLCV C4 specifically interacts with SlTFT2 via its S86 residue, leading to the reprogramming of SlTFT2 subcellular localization and a reduction in its nuclear accumulation. This represents a conserved viral strategy for suppressing host defence, wherein the virus disrupts 14‐3‐3 dependent nucleocytoplasmic signalling, thereby impairing the immune‐related functions executed by the host within the nucleus and ultimately facilitating viral infection and proliferation. We hypothesize that C4, through an S86‐containing motif, occupies one of the binding grooves of SlTFT2 and interacts with it, thereby decreasing nuclear accumulation of SlTFT2 and blocking its ability to interact with defence‐associated partners such as kinases or transcription factors. Future studies will focus on identifying the interaction partners of 14‐3‐3 proteins and verifying whether C4 interferes with the formation of these complexes. Addressing these questions may provide insights for designing antiviral resistant 14‐3‐3 variants, opening new avenues for crop protection.

## 4 Experimental Procedures

### 4.1 Plant Growth, Agroinfiltration and Virus Inoculation

*Nicotiana benthamiana* and tomato ( *Solanum lycopersicum* ‘Moneymaker’) plants were grown in controlled growth chambers at 26°C under white light with a 16 h light/8 h dark photoperiod. Plants at the four‐ to five‐leaf stage were infiltrated with *A. tumefaciens* cultures carrying infectious clones of the TYLCV Jiangsu isolate (GenBank accession GU111505), including the full length TYLCV clone and the C4 mutant infectious clones TYLCV C4S86A and TYLCV C4S86L.

### 4.2 Plasmid Construction

All open reading frames (ORFs) were amplified from tomato cDNA or the TYLCV infectious clone using gene‐specific primers with appropriate restriction sites or homologous recombination arms. All primer sequences and the corresponding restriction sites/recombination arms are listed in Table S1. The PCR products were first cloned into the pMD18‐T vector (TaKaRa) and verified by Sanger sequencing. The verified fragments were then subcloned into the indicated binary vectors by either restriction enzyme digestion and ligation or homologous recombination using the ClonExpress MultiS One Step Cloning Kit (Vazyme), according to the manufacturer's instructions. For constructs generated by single‐enzyme digestion, the linearized vectors were dephosphorylated with alkaline phosphatase prior to ligation to reduce self‐ligation.

For the LCI assay, full‐length coding sequences were cloned into pCAMBIA‐DC‐nLUC and pCAMBIA‐cLUC‐DC vectors via BP recombination, generating C4‐nLUC, C4^S86A^‐nLUC, C4^S86L^‐nLUC and cLUC‐SlTFT2 constructs.

For the BiFC experiment, full‐length coding sequences were cloned into YFP_N_ and YFP_C_ vectors (kindly provided by Prof. Xiaorong Tao, Nanjing Agricultural University), yielding C4‐YFP_N_, C4^S86A^‐YFP_N_, C4^S86L^‐YFP_N_, and SlTFT2‐YFP_C_.

For the CoIP assay, C4, C4^S86A^ and C4^S86L^ fragments fused with a FLAG tag were cloned into the pCambia1300 vector, producing C4‐FLAG, C4^S86A^‐FLAG and C4^S86L^‐FLAG. SlTFT2 was cloned into pCambia1300‐YFP (kindly provided by Prof. Xiaorong Tao, Nanjing Agricultural University), generating SlTFT2‐YFP.

For Y2H assays, the open reading frames of TYLCV C4, SlTFT2, truncated C4 fragments (1–84 aa and 85–97 aa) and the 14‐3‐3 domain of SlTFT2 were amplified and inserted into pGADT7 and pGBKT7 vectors, generating C4‐AD, C4_1–84_‐AD, C4_85–97_‐AD, SlTFT2‐BK and 14‐3‐3 domain‐BK constructs.

For VIGS, a 300‐nt fragment of SlTFT2 was amplified and cloned into pTRV2 to generate pTRV2:SlTFT2. pTRV2, pTRV1, pTRV2‐GUS and pTRV2‐PDS were kindly provided by Prof. Xiaorong Tao, Nanjing Agricultural University.

For overexpression assays, the ORFs of C4 and SlTFT2 were cloned into the PVX vector, producing PVX‐C4 and PVX‐SlTFT2. For transient overexpression, the SlTFT2 ORF was cloned into the pCambia2300:35S × 3FLAG vector (3FLAG), which was kindly provided by Prof. Xiaorong Tao, Nanjing Agricultural University (Feng et al. 2019), yielding 3FLAG‐SlTFT2.

For subcellular localization analysis, the full length open reading frame (ORF) of C4 was amplified, and PCR‐based site‐directed mutagenesis was performed to generate the C4S86A and C4S86L variants. All primers used for amplification and mutagenesis are listed in Table S1. The ORFs of C4, C4^S86A^ and C4^S86L^ were inserted into the BamHI site of pCambia1300‐YFP or pCambia1300‐RFP vectors (kindly provided by Prof. Xiaorong Tao, Nanjing Agricultural University), generating C4‐YFP, C4^S86A^‐YFP, C4^S86L^‐YFP, C4‐RFP, C4^S86A^‐RFP and C4^S86L^‐RFP. Primers used for plasmid construction are listed in Table S1.

### 4.3 Nucleic Acid Extraction and Reverse Transcription

Total RNA was extracted from tomato leaf tissues using TRIzol reagent (TransGEN). Total genomic DNA was isolated from tomato leaf tissues with a plant genomic DNA extraction kit (CWBIO). For each treatment, three independent biological replicates were set, and leaf tissues harvested from six to eight uniformly grown tomato plants were pooled per biological replicate to minimize phenotypic differences among individual plants. The extracted RNA was reverse‐transcribed into cDNA using an ABClone reverse transcription kit for subsequent transcript quantification.

### 4.4 qPCR Analysis

Quantitative PCR was performed using the FastSYBR kit (CWBIO) on a Bio‐Rad CFX Opus 96 system, with three technical replicates for each sample. The tomato *SlActin* gene was used as an internal reference. Primer sequences are provided in Table S1. All RT‐qPCR and qPCR experiments were conducted using three independent biological replicates, and each replicate comprised pooled tissues from 6 to 8 tomato plants.

### 4.5 Fluorescence Microscopy

*Agrobacterium* transformed with SlTFT2‐YFP, C4‐YFP, C4^S86A^‐YFP and C4^S86L^‐YFP was mixed with the *Agrobacterium* transformed with H2B‐RFP to infiltrate *N. benthamiana*. After 2 days of infiltration of *N. benthamiana* leaves, a confocal laser‐scanning microscope was used for imaging. For YFP detection, excitation was set at 488 nm with emission collected at 490–540 nm. For RFP detection, excitation was set at 561 nm with emission collected at 600–650 nm. Images were acquired using an LSM 710 confocal laser‐scanning microscope (Zeiss) and processed with ZEN 2011 software.

### 4.6 LCI Assay

LCI assays were performed as previously described (Wang et al. 2021). *Agrobacterium* carrying C4nLUC, C4^S86A^‐nLUC or C4^S86L^‐nLUC and cLUC‐SlTFT2 plasmids was co‐infiltrated into about 4‐week‐old *N. benthamiana* leaves in a 1:1 ratio. After 2 days, the leaves were sprayed with D‐luciferase potassium salt substrate (Beyotime) on the abaxial surface and images were collected.

### 4.7 BiFC Assay

Recombinant plasmids were transformed into *A. tumefaciens* GV3101. Single colonies were cultured in Luria Bertani (LB) medium containing appropriate antibiotics at 28°C with shaking until the OD₆₀₀ reached 0.6–0.8. Cells were harvested and resuspended in MMA buffer (10 mM MES, 10 mM MgCl₂, 100 μM acetosyringone, pH 5.6), and the final OD₆₀₀ was adjusted to 1.0. Prior to infiltration, *Agrobacterium* suspensions carrying different constructs were mixed in equal volumes.

Healthy 4–5‐week‐old *N. benthamiana* plants were used for infiltration. Plants were maintained at 22°C under a 16 h light/8 h dark photoperiod, and fluorescence signals were observed 48 h after infiltration.

### 4.8 Western Blot and Co‐IP Assays

Co‐IP assays were performed as previously described (Zhao et al. 2018). Total proteins were extracted from 1 g leaf tissue using 2 mL RIPA lysis buffer (Beyotime) supplemented with PMSF and a protease inhibitor cocktail. Protein extracts were incubated with FLAG Trap beads (Beyotime), and immunoprecipitated proteins were eluted in 5× SDS–PAGE loading buffer, boiled for 10 min, and centrifuged at 12,650 *g* for 5 min at 4°C. Proteins were analysed by western blotting.

Membranes were probed with antibodies against FLAG, GFP, dsRed, histone H3 and actin. A mouse polyclonal antibody against TYLCV V1 was kindly provided by Prof. Xueping Zhou (Zhejiang University, China).

### 4.9 TRV‐Mediated Gene Silencing Assays

VIGS assays were performed using TRV‐based vectors. A 300 bp specific fragment of SlTFT2 was selected using the SGN VIGS online tool and inserted into the pTRV2 vector. pTRV1 and pTRV2 constructs were transformed into *A. tumefaciens* GV3101 and co‐infiltrated into three‐leaf‐stage tomato plants. Plants were subsequently inoculated with TYLCV infectious clones at 14–18 dpi. Upper leaves were collected at 14 and 21 dpi for qPCR analysis.

### 4.10 Gene Knockout Assay

The *SlTFT2* sequence was submitted to BioRun Biotechnology Co. Ltd. (Wuhan, China) for generation of knockout constructs in the Micro‐Tom tomato cultivar. The coding regions of *SlTFT2* were cloned into the pHSbdcas9i‐tRNA vectors to obtain plasmids CRISPR‐SlTFT2. Wild‐type and CRISPR‐SlTFT2 plants were inoculated with TYLCV, and upper leaves were collected at 18 and 27 dpi for qPCR analysis.

### 4.11 Gene Overexpression Assays

For overexpression assays, PVX and PVX‐SlTFT2 constructs were introduced into *A. tumefaciens* GV3101 and infiltrated into four‐leaf‐stage tomato plants. At 6–8 dpi, plants were inoculated with TYLCV infectious clones. Upper leaves were collected at 14 and 21 dpi for qPCR analysis.

For transient overexpression, 3FLAG and 3FLAG‐SlTFT2 constructs were co‐infiltrated with TYLCV into *N. benthamiana* leaves. Samples were collected at 48 h for western blot and qPCR analyses using antibodies against FLAG and TYLCV V1.

### 4.12 Y2H Assay

Y2H assays were performed using the Rapid Yeast Competent Cell Preparation Kit (Zoman) according to the manufacturer's instructions. Yeast Y2H strain was cultured on yeast‐peptone‐dextrose agar (YPDA) for 4 days to prepare competent yeast cells. The successfully constructed AD and BK recombinant plasmids containing all or part of the coding regions of C4, C4^S86A^, C4^S86L^ and SlTFT2 were transferred into Y2Hcompetent cells and cultured on SD/−Trp/−Leu and SD/−Trp/−Leu/−His/−Ade media. Interaction was observed after 3–5 days of incubation at 30°C under dark conditions.

### 4.13 Construction of TYLCV‐JS (S86A and S86L) Infectious Clones

To generate infectious clones of the TYLCV‐JS isolate (GU111505) carrying the C4S86A or C4S86L mutation, a two‐step cloning strategy was adopted.

The full length viral genome fragment (designated as the 1A fragment) was amplified and cloned into a TA cloning vector, generating the TA‐TY‐1A construct. Site‐directed mutagenesis was then performed on this construct to introduce the S86A or S86L mutation into the C4 gene, yielding TA‐TY‐1A (C4S86A) and TA‐TY‐1A (C4S86L).

A truncated version of the viral genome (0.8× unit, corresponding to the region from SacI to BamHI within the 1A fragment) was released from a separate 1A fragment by double digestion with SacI and BamHI, and cloned into the binary vector pBinPLUS. The resulting recipient vectors were named pBinPLUS‐0.8A (C4S86A) and pBinPLUS‐0.8A (C4S86L).

The full length 1A fragment carrying the corresponding mutation was released from TA‐TY‐1A (C4S86A/S86L) by BamHI digestion and ligated into the BamHI‐linearized and dephosphorylated pBinPLUS‐0.8A (C4S86A/S86L) recipient vectors, reconstituting a 1.8× unit tandem repeat of the viral genome. The resulting infectious clones were named pBinPLUS‐TYLCV (C4S86A)‐1.8A and pBinPLUS‐TYLCV (C4S86L)‐1.8A.

### 4.14 Nuclear–Cytoplasmic Fractionation Assay

Nuclear and cytoplasmic proteins were isolated using the BB‐3169 kit (BestBio) according to the manufacturer's protocol. Briefly, fresh leaf tissue (200–500 mg) was homogenized in Extraction Buffer A, filtered and centrifuged to separate nuclear and cytoplasmic fractions. Nuclear proteins were extracted using Buffer B, and cytoplasmic proteins were collected following Buffer C treatment. Protease inhibitors were included in all steps.

## Author Contributions

**Danna Gao:** investigation, validation, data curation, software, writing – original draft. **Feng Sun:** writing – review and editing, data curation, visualization. **Xiaoxiao Gao:** investigation. **Yinghua Ji:** funding acquisition, conceptualization, project administration, resources, supervision, writing – review and editing. **Yuelin Zhu:** funding acquisition, conceptualization, supervision, project administration.

## Funding

This work was financially supported by the National Key R&D Program of China (2022YFD1401200) and the National Natural Science Foundation of China (32072506).

## Conflicts of Interest

The authors declare no conflicts of interest.

## Supporting information


**Table S1:** DNA primers used in this study.

**Table S2:** Sequence information of the tomato 14‐3‐3 family and its *Nicotiana* homologues.

**Figure S1:** Partial candidate interactors of TYLCV C4 identified by luciferase complementation imaging (LCI) screening in *Nicotiana benthamiana* and tomato. Candidates marked in red represent the target protein investigated in the present study, and candidates marked in green indicate other putative interactors obtained from this LCI screening. Representative luminescence images show partial potential interactors of TYLCV C4.

**Figure S2:** Photographs of tomato plants inoculated with PVX and PVX‐C4 at 14 days post‐inoculation.

**Figure S3:** Sequence alignment and expression analysis of 14‐3‐3 family members in TRV‐SlTFT2‐silenced tomato plants. (a) Nucleotide sequence alignment between the virus‐induced gene silencing (VIGS) fragment (52–352 bp) of *SlTFT2* and the corresponding region of *SlTFT3*. (b) Relative transcript levels of *SlTFT* family genes in TRV‐SlTFT2‐silenced tomato plants. Note that *SlTFT11*, *SlTFT12* and *SlTFT13* exhibited extremely low transcript abundance and were therefore not included in this reverse transcription‐quantitative PCR analysis.

**Figure S4:** Symptoms of tomato plants that were inoculated TRV:GUS and TRV:SlTFT2 at 17 days post‐inoculation.

**Figure S5:** Gene knockout (CRISPR/Cas9) experiment was performed on tomato (microTom) and CRISPR‐SlTFT2 plasmid was obtained.

**Figure S6:** Tomato plants overexpressing *SlTFT2* showed attenuated symptoms at 8 days post‐inoculation.

**Figure S7:** Identification of critical amino‐acid sites required for C4 interaction with SlTFT2. (a) Schematic diagram showing the strategy for screening key sites responsible for C4‐SlTFT2 interaction within the 84–97 amino acid region of C4. Two serine residues (S86 and S95) were selected and mutated to alanine (S86A, S95A). (b) Luciferase complementation imaging (LCI) assay verifying the interaction between SlTFT2 and C4^S95A^. Luminescence signals indicate that the C4^S95A^ does not disrupt the C4‐SlTFT2–interaction.

**Figure S8:** Co‐localization analysis of SlTFT2 with C4 and C4 mutants. Circles indicate the nuclear regions showing SlTFT2‐YFP fluorescence signals in the merged images. Bar: 20 μm.

## Data Availability

All relevant data are available within the article and its Supporting Information.
